# Transactions of the American Medical Association

**Published:** 1865-02

**Authors:** 


### Book Notices.

Transactions of the American Medical Association. Vol. XV. Philadelphia: Printed for the Association, for 1864.

The Annual volume of Transactions for 1864, though smaller than many of the earlier volumes, is published in excellent style, and contains much matter of interest to the profession. Besides the ordinary record of the Proceedings of the Meeting in New York, in June, 1864, the Code of Ethics, and the full list of Members, the volume contains the following reports and papers:

Death from Air in the Circulation, introduced through the Uterine Sinuses. By H. O. Hitchcock, M.D., of Michigan.

A Modified Ring Pessary for the Treatment and Cure of

Anteflexion and Anteversion of the Uterus. By H. O. Hitchcock, M.D., of Michigan.

Report on the Use of Pessaries. By Augustus K. Gardner, M.D., of New York. With Illustrations.

A Paper on the Relations of Female Patients to the Hospitals for the Insane; the Necessity, on their Account, of a Board of Consulting Physicians to every Hospital. By Horatio R. Storer, M.D., of Boston.

Report on the Practical Working of the U.S. Drug Law. By Edward R. Squibb, M.D., of Brooklyn, N.Y.

On the Naturalization of Cinchonæ on the Eastern Continent. By J. Macgowan, M.D.

Report on Compulsory Vaccination. By James F. Hibbard, M.D., of Indiana.

Report on Military Hygiene. By E. Andrews, M.D., of Ill.

On the Physiological and Dietetic Relations of Phosphorous. By John H. Griscom, M.D., of New York.

Report on the Medical Topography and Epidemic Diseases of the State of Rhode Island. By Charles W. Parsons, M.D., of Providence, R. I.

Report on the Mortality of the City of New York. By Cyrus Ramsey, M.D., of New York.

On the Causes tending to Promote the Extinction of the Aborigines of America. By Jonathan Kneeland, M.D., of N.Y.

On the Pathology of the Lateral Curvature of the Spine. By Charles F. Taylor, M.D., of New York.

Laryngeal Probe Syringe. By R. Macfarlan, M.D., of N.Y.

On Iridectome. By Louis Elsburg, M.D., of New York.

Report on the Treatment of Congenital Fissure of the Palate by Mechanical Appliances. By Norman W. Kingsley, M.D., of New York.

Report on Puerpural Tetanus. By D. L. McGugin, M.D., of Iowa.

Prize Essay.—On the Pathology of Jaundice. By S. Fleet Speir, M.D., of Brooklyn, N.Y.

It will be seen from this list of contents, that the volume contains a considerable variety of topics, all of which are ably discussed, and present matter of interest to the whole profession. The principal defect in the volume, is the inadequate reports of the doings of the several Sections. It is in these sub-organizations that all the scientific work of the Association is done. In them the reports and papers are read, examined, and the questions which they suggest are discussed. Each of them should be supplied with a secretary or reporter capable of keeping an accurate, though condensed, record of all the debates, as well as the mere votes. Such record would not only preserve the important facts, suggestions, and opinions developed in the discussion of the several papers read, which would often be of far more value than the papers themselves, but they would greatly add to the inducements calculated to bring the best men in the profession into active participation in the doings of those Sections. We trust that measures will be taken to secure a much more efficient organization of the several Sections at the coming meeting, than at any of the previous ones.

